# Factors associated with personal and professional beliefs about vaccines: a cross-sectional study of national dental practice-based research dentists

**DOI:** 10.3389/froh.2026.1905870

**Published:** 2026-08-28

**Authors:** David M. Mosen, Jeffrey L. Fellows, Mary Ann McBurnie, Ning Smith, Michael C. Leo, Phillip Crawford, Veerasathpurush Allareddy, Inga Gruß, Dea Papajorgji-Taylor, Gregg H. Gilbert, for the National PBRN Collaborative Group

**Affiliations:** 1 Kaiser Permanente Center for Health Research, Portland, OR, United States; 2 Department of Orthodontics, University of Illinois Chicago College of Dentistry, Chicago, IL, United States; 3 Department of Clinical and Community Sciences, School of Dentistry, University of Alabama at Birmingham, Birmingham, AL, United States

**Keywords:** beliefs, health services research, oral health, public health, surveys and questionnaires, vaccines

## Abstract

**Introduction:**

Our study examined associations between dentists' demographic and practice characteristics and their personal and professional beliefs about vaccines, vaccine delivery and recommendations to influence patient vaccination.

**Methods:**

A cross-sectional survey of 537 dentists in the National Dental Practice Based Research Network. Personal beliefs were assessed using the 5C Scale of Vaccine Hesitancy; professional beliefs were based on dentists' perceived role in recommending and delivering vaccines. Factor analysis was used to construct three outcome measures: 1) personal beliefs about vaccines, 2) professional beliefs about vaccine delivery and 3) professional beliefs about the efficacy of dentist recommendations to influence patient vaccination.

**Results:**

Regarding *personal* beliefs, dentists' age (55–64) was associated with higher support for vaccines; dentists' Hispanic ethnicity was associated with lower support; and a private practice, group setting or dental school was associated with higher support than a private practice, solo setting. Regarding *professional* beliefs, dentists' African-American race was associated with more positive beliefs about vaccine delivery and provider recommendations to influence vaccination. Dentists in dental school and public health settings had higher positive beliefs regarding vaccine delivery; those in rural locations had lower positive beliefs. Dentists treating children and adults reported lower positive beliefs about provider recommendations. Higher *personal support* for vaccines predicted more positive scores on both *professional belief* measures.

**Discussion:**

We identified factors associated with dentists' personal and professional beliefs about vaccines. Future research should assess whether interventions to change dentists' personal beliefs about vaccines also result in changes in professional beliefs and patient vaccination.

## 1 Introduction

Dentists have the potential to expand the delivery of public health services by providing primary healthcare services such as screening, referrals and interventions, including those for tobacco use, hypertension, diabetes, substance use, and mental health (1–4). Dentists have also contributed to disease prevention by providing vaccinations to prevent influenza, HPV, and COVID-19 (5–7). Administration of vaccines in dental practice settings has been championed by the American Dental Association and other oral health leaders and organizations (8, 9). Dental patients, who may see their dental care providers more frequently than their primary care medical providers, tend to be receptive to vaccine recommendations and administration by dentists, although receptivity has been shown to vary by vaccine type (10, 11). Dentists' beliefs about vaccines also vary, including both their personal beliefs (willingness to accept vaccinations for themselves), and their professional willingness to recommend and administer vaccinations to their patients. Although a substantial proportion of dentists have reported being willing to administer vaccines, vaccine delivery in dental practices remains rare (12–14). Some barriers to more-comprehensive vaccine delivery in dental practices have been identified, including lack of sufficient knowledge/training, state and federal regulations, and reimbursement considerations (15). Little is known, however, about how dentists' personal and professional beliefs might impact their willingness to recommend and deliver vaccines to their patients. Dentists' personal and professional beliefs regarding vaccines may impact not only dentists' willingness to recommend and deliver vaccines, but also patients' willingness to receive vaccines.

Herein we report results from a survey of dentists who are members of the National Dental Practice-Based Research Network (Network), examining personal and professional beliefs about vaccines and vaccine delivery. Network practitioners represent a broad national cross-section of dentists working in a variety of practice settings and serving a diverse patient population (16, 17). Our study objectives were two-fold. First, examine the association of dentists' demographic and practice characteristics with their *personal* beliefs regarding vaccines and their *professional* beliefs regarding a) vaccine delivery and b) efficacy of dental provider recommendations to influence receipt of vaccines. Second, examine the association of dentists' personal beliefs regarding vaccines with both professional belief measures. Understanding key factors associated with dentists' beliefs about vaccines will inform strategies to improve vaccine uptake among their patients.

## 2 Methods

### 2.1 Design

We used a cross-sectional questionnaire design to obtain information about active U.S. dentists' personal and professional beliefs about vaccines and vaccine delivery. Responses were combined with data from the Network's Enrollment Questionnaire (EQ) (18); maintained by the Network Coordinating Center (17). The study conforms to STROBE guidelines.

### 2.2 Setting

The study was conducted by the Network, a consortium of over 9,000 dentists, dental hygienists, dental office personnel, clinical researchers, and other stakeholders, working to improve oral health care through the conduct and dissemination of dental research (16). Network practitioners are assigned to one of 6 regional nodes. Each Network regional node's institutional review board (IRB) reviewed and approved all study materials and procedures.

### 2.3 Participants

To be eligible, participants had to be enrolled in the Network and have updated profile information after June 2019, indicate willingness to participate in questionnaire studies, and be an active U.S. general or pediatric dentist, or dental public health specialist providing primary dental care. Network staff extracted email and telephone numbers for 2,079 eligible dentists for recruitment outreach from a centralized practitioner database; demographic and practice characteristics data were also extracted for analysis and to manage respondent representativeness if necessary.

### 2.4 Recruitment

Available funding for the project restricted surveys to about 500 participants. Beginning on 1/6/2022, the recruitment plan included an initial email invitation and a 1-week reminder, each with personalized REDCap survey links sent by the Network staff to all eligible dentists, followed by targeted email and telephone outreach by regional node staff for subgroups that responded at rates lower than the eligible population. However, recruitment goals were reached within 19 days of recruitment (1/6/2022-1/24/2022) by initial e-mail and 1-week reminder and no further outreach was needed. Practitioners were offered $50 for completing the questionnaire.

### 2.5 Study data

The survey included questions about participant practice settings and personal and professional beliefs about vaccines. We obtained practitioner demographics (age, gender, race, ethnicity) and Network region from the EQ. We report demographic and practice characteristics for study participants and for all eligible dentists in the Network.

Participants indicated their primary practice setting [private solo practice, private small group practice (2–4 dentists), private large group practice (≥5 dentists), managed care, preferred provider organization, corporate practice, academic/dental school, hospital, community health practice, public health practice, federal practice, or military practice), as well as practice location (rural, suburban, or urban), if the practice was in a health professional shortage area (HPSA) (19), and whether their patients were primarily children (age < 18 years), adults, or both.

Dentists' personal beliefs towards vaccines were assessed using seven items from the 5C Scale of Vaccine Hesitancy, a valid and reliable measure: three items measuring confidence in efficacy of vaccines and one item each assessing complacency, constraints, calculation, and collective responsibility (20). Professional beliefs regarding dentists' roles in vaccine screening and delivery were measured using nine items developed by the study team. These questions were developed by the study team and Network Practitioner Executive Committee using the “Think-Aloud” method (21, 22). All responses were measured on a 5-point Likert scale (strongly disagree to strongly agree).

### 2.6 Statistical analysis

SAS 9.4 (Cary, NC) was used for all analyses in the study. Because this study was exploratory, examining multiple associations without a single primary outcome, we did not perform an *a priori* power analysis. Instead, we aimed to maximize the sample size within available funding. We first conducted descriptive analysis of dentists' demographic and practice characteristics (including non-respondent analysis), seven items describing personal beliefs about vaccines, and nine items representing dentists' professional beliefs about vaccine delivery and recommendations. We then used exploratory factor analysis (EFA) to reduce the number of potential dependent variables and simplify regression modelling. EFA enabled efficient grouping of the individual variables representing professional beliefs about vaccines. Lastly, multivariable linear models were constructed to test the associations between: 1) dentists' demographic and practice characteristics (predictor measures) and personal beliefs about vaccines (outcome measure 1); 2) dentists' demographic and practice characteristics (predictor measures), personal beliefs about vaccines (predictor measure) and professional beliefs about vaccine delivery (outcome measure 2); and 3) dentists' demographic and practice characteristics (predictor measures), personal beliefs about vaccines (predictor measures) and professional beliefs about efficacy of dentist recommendations to influence patient receipt of vaccines (outcome measure 3). Multivariable linear models were run for all participants who did not have missing data for predictor and outcome measures (*N* = 511). Those with missing data (<5%) were excluded. For the multivariable analysis, we report regression coefficients, 95% confidence intervals (CIs) and *p*-values for each model.

#### 2.6.1 Factor analysis to identify outcome variable 1 (personal beliefs about vaccines, 4C scale)

Details regarding the factor analysis are presented in Supplementary Table S1. Based on the factor analysis, a unit-based “*4C scale*” score was constructed representing the mean of the complacency, constraints, confidence, and collective responsibility measures as a measure of personal support for vaccines (six items total). The calculation measure (weighing benefits and risks) showed no communality with other measures and was not included in the measure. We reverse-scored the measures for complacency and constraints so that higher mean 4C scale scores reflect greater personal support for vaccinations. A complete description of the personal beliefs questions about vaccines is provided in Appendix A along with the components of the 5C Scale (e.g., complacency, etc.).

#### 2.6.2 Factor analysis to identify outcome variable 2 (professional beliefs about vaccine delivery)

Factor analysis yielded two factors regarding professional beliefs about vaccine delivery. The first factor included seven items identifying a theme of beliefs about *dental practitioners providing vaccines*. A higher score on this measure indicates greater support for dentists to provide vaccines. A description of these seven questions is provided in Appendix B.

#### 2.6.3 Factor analysis to identify outcome variable 3 (professional beliefs regarding the efficacy of dental provider recommendations to influence patient receipt of vaccines)

The second factor included two items identifying a theme of *dental practitioners' beliefs regarding the efficacy of dental providers to influence patient receipt of vaccines*. A description of these 2 questions is provided in Appendix B. A higher score on this measure indicates a stronger belief that dental provider recommendations of vaccines are effective in influencing patient receipt of vaccines.

## 3 Results

### 3.1 Population characteristics

We identified 2,079 dentists from the Network's practitioner enrollment database who were eligible to participate as of January 4, 2022. A total of 537 dentists completed the questionnaire between January 6–24, 2022, for a response rate of about 25%. Respondents had a mean age of 51.3 years; 38% were female, 5% Hispanic/Latino, and 70% non-Hispanic White person(s) with most practicing as general dentists (Table 1). Fifty percent reported their patients were primarily adults, while 44% reported serving patients of all ages. Respondents predominantly practiced in private practice settings (77%) and were located in suburban (56%) and urban (29%) areas. Fifteen percent reported practicing in a Health Professional Shortage Area (HPSA). Calculated standardized meaningful differences (SMDs) were less than 0.20 between survey respondents (*N* = 537) compared to non-respondents (*N* = 1,542) with respect to age, sex, race, practice type, practice setting and region, suggesting minimal differences between the two groups (23) (Table 1).

**Table 1:** Demographic and practice characteristics for survey respondents and non-respondents among eligible network dentists.

Dental Characteristic	Respondents	Non-Respondents	Standardized Mean Difference (SMD)
	*N* = 537	*N* = 1,542	
Age, mean +/- SD	51.3 +/- 12.8	52.0 +/- 13.1	0.06
Female	202 (38%)	580 (38%)	0.0001
Race-ethnicity			
Hispanic	26 (5%)	113 (7%)	0.15
Asian	63 (12%)	178 (12%)	
Black/African-American	27 (5%)	91 (6%)	
Other/unknown	46 (9%)	173 (11%)	
Non-Hispanic White person(s)/people	375 (70%)	987 (64%)	
Practice type			
General dentist	511 (95%)	1,450 (94%)	0.05
Pediatric dentist/public health dentist	26 (5%)	92 (6%)	
Primary patient population			
Adults	268 (50%)	NA^a^	NA
Children and adolescents (<18 years)	33 (6%)	NA^a^	
Both	236 (44%)	NA^a^	
Practice setting			
Private, solo	226 (42%)	641 (42%)	0.11
Private, group	188 (35%)	511 (33%)	
MCO/PPO/corporate	50 (9%)	159 (10%)	
Academic/hospital	23 (4%)	86 (6%)	
CHC/public health/federal/military	50 (9%)	145 (9%)	
Network region			
Midwest	72 (13%)	174 (11%)	0.19
Northeast	102 (19%)	225 (15%)	
South Atlantic	92 (17%)	257 (17%)	
South Central	75 (14%)	240 (16%)	
Southwest	106 (20%)	397 (26%)	
Western	90 (17%)	249 (16%)	
Practice location			
Urban	155 (29%)	NA^a^	NA
Suburban	299 (56%)	NA^a^	
Rural	83 (15%)	NA^a^	
Health Professional Shortage Area			
Yes	83 (15%)	NA^a^	NA
No	377 (70%)	NA^a^	
Don’t know	77 (14%)	NA^a^	

MCO, managed care organization; PPO, preferred provider organization; CHC, community health center. Some categories had missing responses and were excluded.

a Information was not available (NA) for the eligible non-respondents. Some column percentages may not equal 100 due to rounding.

### 3.2 Descriptive statistics: personal beliefs about vaccines

Dentists reported positive personal beliefs regarding vaccines. Most dentists agreed/strongly agreed that vaccines were safe (86%), effective (90%), and felt public authorities' decisions were in the best interest of the community (69%). There was a strong sense of community responsibility as 92% disagreed/strongly disagreed that they didn't need to get vaccinated if everyone in the community is vaccinated, and 91% agreed/strongly agreed that they weigh risks and benefits of their decision to get vaccinated. Nearly all (94%) disagreed/strongly disagreed that vaccination was unnecessary because vaccine-preventable diseases were uncommon, while 90% disagreed/strongly disagreed that everyday stress prevented them from getting vaccinated (see Appendix A).

### 3.3 Descriptive statistics – professional beliefs about vaccines

Results were more mixed regarding professional beliefs about vaccines. Most agreed/strongly agreed that dentists can have an important role in the public health effort to reduce the spread of infectious disease. Nearly two-thirds agreed or strongly agreed that practitioners should document patients' vaccine status and provide vaccines either at their dental offices or in community settings. Over 90% strongly agreed/agreed that dental practitioners could provide vaccines at dental offices if provided support and resources. Most strongly agreed/agreed that dental practitioners could increase vaccination rates in the community (70%) and help reduce the burden of vaccine delivery on other providers (77%). Most strongly agreed/agreed (91%) that it was very important for dental office staff to be vaccinated. However, only 57% strongly agreed/agreed that patients were more likely to get vaccinated because their dentist was vaccinated and only 42% strongly agreed/agreed that patients would be more likely to get vaccinated if it was recommended by their dental practitioner (see Appendix B).

#### 3.3.1 Multivariable regression results: personal beliefs regarding vaccines

Regression modeling showed significant associations between 4C scale score and age group, race/ethnicity, and practice setting. 4C scale scores were 0.17 higher among dentists ages 55–64 (CI: 0.01, 0.32) than dentists age ≥ 65 years, while scores among Hispanic dentists were 0.31 lower (CI: −0.56, −0.07) than non-Hispanic White dentists. 4C scale scores were 0.27 higher for a dental school setting (CI: 0.005, 0.54) and 0.14 higher for a private practice, group setting (CI: 0.03, 0.26) than a private practice, solo setting (Table 2).

**Table 2:** Multivariable results: association of dentists' demographic and practice characteristics with personal beliefs regarding vaccines (4C score).

Dental Characteristic	*N*	Regression Coefficient	95% CI	*p*-value
Age group				
27–34	56	0.01	(−0.18, 0.21)	NS^a^
35–44 years	120	0.02	(−0.15, 0.18)	NS^a^
45–54 years	111	0.08	(−0.09, 0.24)	NS^a^
55–64 years	130	0.17	(0.01, 0.32)	0.032
≥65 years	94	ref	ref	NA^b^
Gender				
Female	189	0.02	(−0.09, 0.12)	NS^a^
Male	322	ref	ref	NA^b^
Race-ethnicity				
Hispanic	25	−0.31	(−0.56,−0.07)	0.011
Asian	59	−0.13	(−0.30, 0.04)	NS^a^
Black/African American	24	0.14	(−0.11, 0.38)	NS^a^
Other/unknown	40	−0.12	(−0.31, 0.07)	NS^a^
Non-Hispanic White person(s)/people	363	ref	ref	NA^b^
Practice setting				
Private practice, solo	215	ref	ref	
Private practice, group	182	0.14	(0.03, 0.26)	0.015
MCO/PPO/corporate	47	0.16	(−0.03, 0.34)	NS^a^
Dental school/hospital	21	0.27	(0.005, 0.54)	0.046
CHC/pub. hlth./federal/military	46	0.12	(−0.08, 0.33)	NS^a^
Primary patient population				
Children (≤18) or all ages	254	0.00	(−0.10, 0.10)	NS^a^
Adult patients only	257	ref	ref	NA^b^
Network region				
Midwest	68	−0.03	(−0.21, 0.16)	NS^a^
Northeast	100	0.02	(−0.16, 0.19)	NS^a^
South Atlantic	88	−0.12	(−0.30, 0.06)	NS^a^
South Central	72	−0.17	(−0.35, 0.02)	NS^a^
Southwest	100	−0.06	(−0.23, 0.10)	NS^a^
Western	83	ref	ref	NA^b^
Practice location				
Urban	144	−0.05	(−0.17, 0.07)	NS^a^
Rural	81	0.08	(−0.08, 0.23)	NS^a^
Suburban	286	ref	ref	NA^b^
Health Professional Shortage Area				
Yes	78	0.01	(−0.16, 0.18)	NS^a^
Don't know	76	−0.02	(−0.16, 0.13)	NS^a^
No	357	ref	ref	NA^b^

MCO, managed care organization; PPO, preferred provider organization; CHC, community health center. Some categories had missing responses and were excluded.

a NS = not significant at *p* < 0.05.

b NA = not applicable.

#### 3.3.2 Multivariable regression results: professional beliefs regarding vaccine delivery

Regression modeling showed significant associations between professional beliefs regarding vaccine delivery and race/ethnicity, practice setting, practice location and personal belief scores regarding vaccines. Scores on the factor related to vaccine delivery were 0.28 points higher for African-American dentists (CI: 0.02–0.55) dentists than for non-Hispanic White dentists. In terms of practice setting, scores on beliefs towards vaccine delivery were 0.32 points higher for those practicing in dental school/hospital settings (CI: 0.03, 0.60) and 0.42 points higher for those practicing in the CHC/public health/federal/military settings (CI: 0.20, 0.64), compared to those practicing in private practice solo clinics. With respect to clinic location, scores on vaccine delivery were 0.22 points lower for those practicing in rural clinics (CI: −0.39, −0.05) compared to those practicing in suburban clinics. Last, each 1-point increase in 4C Score and Calculation score was associated with a 0.54 point (CI: 0.44, 0.63) and 0.08 point (CI: 0.02, 0.15) increase in the professional beliefs regarding vaccines score, respectively (Table 3).

**Table 3:** Multivariable results: association of dentists' demographic, provider characteristics and personal beliefs regarding vaccines with professional beliefs regarding vaccine delivery.

Dental Characteristic	*N*	Regression Coefficient	95% CI	*p*-value
Age group				
27–34	56	−0.18	(−0.40, 0.03)	NS^a^
35–44 years	120	−0.03	(−0.21, 0.15)	NS^a^
45–54 years	111	0.03	(−0.15, 0.20)	NS^a^
55–64 years	130	−0.06	(−0.22, 0.11)	NS^a^
≥65 years	94	ref	ref	NA^b^
Gender				
Female	189	0.05	(−0.07, 0.16)	NS^a^
Male	322	ref	ref	NA^b^
Race-ethnicity				
Hispanic	25	0.10	(−0.16, 0.36)	NS^a^
Asian	59	0.17	(−0.02, 0.35)	NS^a^
Black/African American	24	0.28	(0.02, 0.55)	0.038
Other	40	0.14	(−0.07, 0.35)	NS^a^
Non-Hispanic White person(s)/people	363	ref	ref	NA^b^
Practice setting				
Private practice, solo	215	ref	ref	NA^b^
Private practice, group	182	0.01	(−0.12, 0.13)	NS^a^
MCO/PPO/Corporate	47	0.19	(−0.02, 0.39)	NS^a^
Dental school/hospital	21	0.32	(0.03, 0.60)	0.031
CHC/pub. hlth./federal/military	46	0.42	(0.20, 0.64)	0.0002
Primary patient population				
Children (≤18) or all ages	254	−0.01	(−0.12, 0.10)	NS^a^
Adult patients only	257	ref	ref	NA^b^
Network region				
Midwest	68	0.06	(−0.14, 0.26)	NS^a^
Northeast	100	−0.02	(−0.21, 0.17)	NS^a^
South Atlantic	88	−0.11	(−0.30, 0.08)	NS^a^
South Central	72	0.04	(−0.16, 0.24)	NS^a^
Southwest	100	−0.10	(−0.28, 0.08)	NS^a^
Western	83	ref	ref	NA^b^
Practice location				
Urban	144	0.03	(−0.10, 0.15)	NS^a^
Rural	81	−0.22	(−0.39, −0.05)	0.010
Suburban	286	ref	ref	NA^b^
Health Professional Shortage Area				
Yes	78	−0.07	(−0.25, 0.11)	NS^a^
Don't know	76	0.02	(−0.14, 0.17)	NS^a^
No	357	ref	ref	NA^b^
Personal beliefs about vaccines				
4C score	511	0.54	(0.44, 0.63)	<0.0001
Calculation	511	0.08	(0.02, 0.15)	0.010

MCO, managed care organization; PPO, preferred provider organization; CHC, community health center. Some categories had missing responses and were excluded.

a NS = not significant at *p* < 0.05.

b NA = not applicable.

#### 3.3.3 Multivariable regression results: professional beliefs regarding the efficacy of dental provider recommendations to influence patient receipt of vaccines

Regression modeling showed significant associations between efficacy of dental provider recommendations to influence patient receipt of vaccines and race/ethnicity, patient population served, and personal beliefs about vaccines. Professional beliefs regarding efficacy of dental provider recommendations to influence patient receipt of vaccines were 0.46 points higher for African-American dentists (CI: 0.11, 0.80) than for non-Hispanic White dentists and 0.15 points lower for those dentists that served children and adults (CI: −0.30, −0.01) than for dentists that served adults only. Last, each 1-point increase in the 4C score was associated with a 0.44-point increase (CI: 0.31, 0.56) in professional beliefs regarding efficacy of dental provider recommendations to influence patient receipt of vaccines (Table 4).

**Table 4:** Multivariable results: association of dentists' demographic, provider characteristics and personal beliefs regarding vaccines with professional beliefs regarding efficacy of provider recommendations to influence receipt of vaccines.

Dental Characteristic	*N*	Regression Coefficient	95% CI	*p*-value
Age group				
27–34	56	−0.09	(−0.37, 0.19)	NS^a^
35–44 years	120	−0.09	(−0.32, 0.14)	NS^a^
45–54 years	111	0.01	(−0.22, 0.24)	NS^a^
55–64 years	130	−0.08	(−0.30, 0.13)	NS^a^
≥65 years	94	ref	ref	NA^b^
Gender				
Female	189	−0.07	(−0.23, 0.08)	NS^a^
Male	322	ref	ref	NA^b^
Race-ethnicity				
Hispanic	25	0.16	(−0.18, 0.50)	NS^a^
Asian	59	0.13	(−0.10, 0.37)	NS^a^
Black/African American	24	0.46	(0.11, 0.80)	0.010
Other/unknown	40	0.07	(−0.19, 0.34)	NS^a^
Non-Hispanic White person(s)/people	363	ref	ref	NA^b^
Practice setting				
Private practice, solo	215	ref	ref	NA^b^
Private practice, group	182	0.06	(−0.10, 0.22)	NS^a^
MCO/PPO/corporate	47	0.03	(−0.24, 0.29)	NS^a^
Dental school/hospital	21	−0.20	(−0.57, 0.18)	NS^a^
CHC/pub. hlth./federal/military	46	0.16	(−0.13, 0.44)	NS^a^
Primary patient population				
Children (≤18) or all ages	254	−0.15	(−0.30, −0.01)	0.036
Adult patients only	257	ref	ref	NA^b^
Network region				
Midwest	68	−0.05	(−0.31, 0.21)	NS^a^
Northeast	100	0.03	(−0.21, 0.28)	NS^a^
South Atlantic	88	−0.14	(−0.38, 0.11)	NS^a^
South Central	72	0.10	(−0.16, 0.36)	NS^a^
Southwest	100	0.001	(−0.23, 0.24)	NS^a^
Western	83	ref	ref	NA^b^
Practice location				
Urban	144	0.06	(−0.11, 0.23)	NS^a^
Rural	81	−0.01	(−0.23, 0.21)	NS^a^
Suburban	286	ref	ref	NA^b^
Health Professional Shortage Area				
Yes	78	0.10	(−0.14, 0.33)	NS^a^
Don't know	76	−0.15	(−0.35, 0.05)	NS^a^
No	357	ref	ref	NA^b^
Personal beliefs about vaccines				
4C score	511	0.44	(0.31, 0.56)	<0.0001
Calculation	511	0.02	(−0.06, 0.10)	NS^a^

MCO, managed care organization; PPO, preferred provider organization; CHC, community health center. Some categories had missing responses and were excluded.

a NS = not significant at *p* < 0.05.

b NA = not applicable.

## 4 Discussion

Most dentists in our study reported high levels of personal support for vaccines. Similarly, most expressed positive professional beliefs toward delivering vaccines in dental settings. However, professional beliefs were lower regarding the perceived effectiveness of dental provider recommendations in influencing patient vaccine uptake and patient willingness to receive vaccines.

This study makes a unique contribution to the dental health services literature by identifying independent factors associated with *personal* beliefs regarding vaccines, adjusting for other key dental practice characteristics. Dental practitioners aged 55–64 had higher personal support for vaccines than dentists aged 65+; Hispanic dentists had lower personal support for vaccines than non-Hispanic White dentists. Moreover, those practicing in a group private practice or dental school/hospital settings had higher personal support for vaccines than those practicing in private practice solo settings.

This study is also one of the first of which we are aware to identify key factors associated with *professional* beliefs regarding vaccine delivery and efficacy of dental provider recommendations to influence patients' willingness to be vaccinated. African-American dentists were more likely to have positive beliefs towards both vaccine delivery and efficacy of dental provider recommendations to influence patient receipt of vaccines. Those practicing in either dental school or public health settings had higher positive beliefs regarding vaccine delivery than those practicing in private practice solo clinics, while those practicing in rural settings had lower positive beliefs regarding vaccine delivery than those practicing in suburban settings. Moreover, dentists in practices that cared for both children and adults were less likely to report positive beliefs regarding the efficacy of dental provider recommendations to influence patient receipt of vaccines than dentists in practices that only cared for adults. Last, and of critical importance, we found that *higher personal support* for vaccines was predictive of more-positive scores on both measures of *professional beliefs* toward vaccines.

Our findings that dentists of Hispanic ethnicity had less favorable personal beliefs toward vaccines, compared to non-Hispanic White dentists, was consistent with prior research findings (24–26). Like our study, previous research also found that dentists practicing in dental school settings had higher personal support for vaccines than dentists in private practice settings (14, 27). However, in contrast to our study findings that found no difference among African-American vs. White dentists regarding personal support for vaccines, prior research found lower personal support among African-American dental practitioners compared to White dental practitioners (24, 26).

Our study makes original contributions to the dental health services literature in two ways. First, the national sample and rigorous statistical methods provide unique insights into key factors associated with dentists' personal and professional beliefs toward vaccines. Second, to our knowledge, this is the first study to examine the association between dentists' personal beliefs and their professional beliefs regarding vaccines.

These results have implications for policy and clinical practice. Namely, they suggest that changing personal beliefs towards vaccines may impact professional beliefs regarding vaccines. Thus, possible interventions to increase personal support for vaccines could also affect professional beliefs towards: 1) vaccine delivery in dental settings and 2) the efficacy of dental providers recommendations to influence patient receipt of vaccines. Moreover, dental providers at rural clinics and those seeing children could be particularly important to target for intervention efforts to change beliefs both towards vaccine delivery and increasing efficacy of dental provider recommendations to influence patient receipt of vaccines, respectively.

This study had important limitations. First, the cross-sectional study design limited our ability to assess causation between factors associated with dentists' personal and professional beliefs towards vaccination. Second, reaching our recruitment within 19 days may have affected the representativeness of the study population even though there were no meaningful differences in observable characteristics with non-respondents. Our respondents may have been more interested in vaccine issues, positive or negative, compared to non-respondents. We also found no meaningful differences in responses between early and late survey completers (results not shown). Third, social desirability bias among dentists may have contributed to reporting of more favorable personal and professional views regarding vaccines. Fourth, sample size limitations may have reduced the ability to detect differences in outcome measures among smaller subgroups (e.g., younger dentists). Last, the measures of professional beliefs regarding vaccines were not validated and further research is needed to validate these measures in other settings. Despite these limitations, the results from the study represent the beliefs of a national sample of active general dentists in the Network.

## 5 Conclusion

Our study identified dentists' demographic and practice characteristics associated with personal and professional beliefs regarding vaccines. Moreover, we found higher personal support for vaccines among dentists was associated with: 1) more positive beliefs about vaccine delivery and 2) more positive beliefs about the likelihood that dental provider recommendations can positively influence patient uptake of vaccines.

Future research should assess whether interventions to change personal beliefs about vaccines also result in changes in professional beliefs regarding vaccines, and ultimately to more receipt of vaccines by patients. In addition, more research is needed to understand whether the associations of provider characteristics with dentists' beliefs regarding vaccines hold for other health care services, such as screening and referral for substance abuse disorder and other mental health conditions.

## Data Availability

The datasets presented in this study can be found in online repositories. The names of the repository/repositories and accession number(s) can be found below: De-identified data for this analysis are available at the Network website at: https://www.nationaldentalpbrn.org/recruiting-ongoing-upcoming-completed/#1744657580672-54050635-2794.

## Appendix A

**Table A1:** Dentists’ personal beliefs about vaccines.

5C Category	Question	Strongly Agree	Agree	Neither Agree or Disagree	Disagree	Strongly Disagree
Confidence	I am confident vaccines are safe. (*N* = 523)	46%	40%	10%	3%	2%
Confidence	Vaccinations are effective. (*N* = 525)	53%	37%	9%	1%	2%
Confidence	Regarding vaccines, I am confident that public authorities decide in the best interest of the community. (*N* = 524)	27%	42%	18%	9%	4%
Complacency	When everyone is vaccinated, I don't have to get vaccinated too. (*N* = 525)	1%	1%	6%	31%	61%
Calculation	When I think about getting vaccinated, I weigh benefits and risks to make the best decision possible. (*N* = 524).	37%	54%	3%	4%	2%
Collective Responsibility	Vaccination is unnecessary because vaccine-preventable diseases are not common anymore. (*N* = 525)	1%	1%	5%	21%	73%
Constraints	Everyday stress prevents me from getting vaccinated. (*N* = 524)	1%	2%	7%	28%	62%

## Appendix B

**Table B1:** Dentists’ professional beliefs about vaccines.

Profess. Beliefs Measure	Question	Strongly Agree	Agree	Neither Agree or Disagree	Disagree	Strongly Disagree
Vaccine Delivery	^a^Screening and documenting patient's vaccination status. (*N* = 527)	25%	41%	18%	12%	3%
Vaccine Delivery	^a^Providing vaccinations outside the dental office through local organizations. (*N* = 526)	19%	46%	25%	7%	3%
Vaccine Delivery	^a^Providing vaccinations at their dental office. (*N* = 526)	19%	44%	23%	10%	4%
Vaccine Delivery	Dental practitioners are capable of providing vaccines at dental offices if given appropriate training, resources, and compensation. (*N* = 525)	47%	46%	5%	1%	1%
Vaccine Delivery	Dental professional-administered vaccines, especially for oral health-related disease like HPV, can have a tremendously positive impact on increasing vaccination rates and improving population health.(*N* = 526)	24%	46%	23%	5%	2%
Vaccine Delivery	Having dentists administer vaccines in their communities can relieve some of the burden on the current vaccine provider network. (*N* = 523)	25%	52%	16%	5%	2%
Vaccine Delivery	It is very important for dental practitioners and staff to be vaccinated against infectious disease. (*N* = 525)	69%	22%	5%	1%	3%
Efficacy of Provider Recommendation to Influence Patient Receipt of Vaccines	Patients are more likely to get vaccinations if they are recommended by their dental care provider. (*N* = 525)	9%	33%	44%	11%	3%
Efficacy of Provider Recommendation to Influence Patient Receipt of Vaccines	Patients are more likely to get recommended vaccinations if they know their dental providers have received these vaccinations. (*N* = 527)	16%	41%	35%	6%	2%

a Each question begins with the stem, “Dental practitioners can have an important role in the public health effort to reduce the spread of infectious disease, including: 1) screening and documenting patient's vaccination status, 2) providing vaccinations outside the dental office through local organizations and 3) providing vaccinations at their dental office.”
