# Supplementary material for: Factors associated with personal and professional beliefs about vaccines: a cross-sectional study of national dental practice-based research dentists
Source: Front Oral Health. 2026 Aug 28;7:1905870. doi: 10.3389/froh.2026.1905870 (PMC13563270; doi:10.3389/froh.2026.1905870)
Supplement: Supplementary file 1 [file Table1.docx]

Supplemental Table 1.

| **Vaccine Belief Measure** | **Description of Factor Analysis** | **Number of Items in Measure** | **Cronbach’s Alpha** |
| --- | --- | --- | --- |
| Measure 1: Personal Beliefs About Vaccines | Principal axis factoring of the seven items of the 5C scales found only one factor with an eigenvalue greater than 1.0 and four with factor loadings ≥.32. The contributions of complacency (factor loading = .88, possible range from -1 to +1) and constraints (factor loading =.65) were positively associated with the factor score while the contributions of confidence (factor loading = -.55) and collective responsibility (factor loading = -.64) were negative. The calculation item (weighing benefits and risks) showed low communality (.07) with other measures and did not load onto a second factor. | Six | .76 |
| Measure 2: Professional Beliefs About Vaccine Delivery | Principal axis factoring and oblimin factor rotation of the nine professional belief questions resulted in two factors with eigenvalues ≥1. The first factor included seven items with loadings between .88 and .39, suggesting a theme of beliefs about *dental practitioners providing vaccines*. | Seven | .87 |
| Measure 3: Professional Beliefs About the Efficacy of Dental Providers to Influence Patient Receipt of Vaccines | The second factor included two items with loadings of .81 each, suggesting a theme of *dental practitioners’ beliefs regarding the efficacy of dental providers to influence patient receipt of vaccines*. | Two | .81 |
